# Ethanol-Induced Transcriptional Activation of Programmed Cell Death 4 (*Pdcd4*) Is Mediated by GSK-3β Signaling in Rat Cortical Neuroblasts

**DOI:** 10.1371/journal.pone.0098080

**Published:** 2014-05-16

**Authors:** Amanjot Kaur Riar, Madhusudhanan Narasimhan, Mary Latha Rathinam, Dhanashree Vedpathak, Srinivas Mummidi, George I. Henderson, Lenin Mahimainathan

**Affiliations:** 1 Department of Pharmacology and Neuroscience, Texas Tech University Health Sciences Center, Lubbock, Texas, United States of America; 2 South Plains Alcohol and Addiction Research Center, Texas Tech University Health Sciences Center, Lubbock, Texas, United States of America; 3 Center for Personalized Medicine, South Texas Veterans Health Care System, and University of Texas Health Science Center at San Antonio, San Antonio, Texas, United States of America; Temple University School of Medicine, United States of America

## Abstract

Ingestion of ethanol (ETOH) during pregnancy induces grave abnormalities in developing fetal brain. We have previously reported that ETOH induces programmed cell death 4 (PDCD4), a critical regulator of cell growth, in cultured fetal cerebral cortical neurons (PCNs) and in the cerebral cortex *in vivo* and affect protein synthesis as observed in Fetal Alcohol Spectrum Disorder (FASD). However, the mechanism which activates PDCD4 in neuronal systems is unclear and understanding this regulation may provide a counteractive strategy to correct the protein synthesis associated developmental changes seen in FASD. The present study investigates the molecular mechanism by which ethanol regulates PDCD4 in cortical neuroblasts, the immediate precursor of neurons. ETOH treatment significantly increased PDCD4 protein and transcript expression in spontaneously immortalized rat brain neuroblasts. Since PDCD4 is regulated at both the post-translational and post-transcriptional level, we assessed ETOH’s effect on PDCD4 protein and mRNA stability. Chase experiments demonstrated that ETOH does not significantly impact either PDCD4 protein or mRNA stabilization. PDCD4 promoter-reporter assays confirmed that PDCD4 is transcriptionally regulated by ETOH in neuroblasts. Given a critical role of glycogen synthase kinase 3β (GSK-3β) signaling in regulating protein synthesis and neurotoxic mechanisms, we investigated the involvement of GSK-3β and showed that multifunctional GSK-3β was significantly activated in response to ETOH in neuroblasts. In addition, we found that ETOH-induced activation of PDCD4 was inhibited by pharmacologic blockade of GSK-3β using inhibitors, lithium chloride (LiCl) and SB-216763 or siRNA mediated silencing of GSK-3β. These results suggest that ethanol transcriptionally upregulates PDCD4 by enhancing GSK-3β signaling in cortical neuroblasts. Further, we demonstrate that canonical Wnt-3a/GSK-3β signaling is involved in regulating PDCD4 protein expression. Altogether, we provide evidence that GSK-3β/PDCD4 network may represent a critical modulatory point to manage the protein synthetic anomalies and growth aberrations of neural cells seen in FASD.

## Introduction

Fetal alcohol spectrum disorder (FASD) is a global health problem. FASD encompasses a gamut of permanent birth defects caused by maternal alcohol consumption during pregnancy affecting 1 in every 100 live births in United States and Europe [1], [2]. The most severe scale of FASD is symbolized by fetal alcohol syndrome (FAS) which is exemplified by facial dysmorphology, aberrations in growth and central nervous system (CNS) impairment. Regardless of widely disseminated knowledge about potential adverse effects of alcohol, a large number of women consume alcohol during pregnancy. Especially important, is that 18% of pregnant women abuse alcohol during their first trimester of pregnancy [3]. The effects of FAS are serious and irreparable and survivors may have to endure life-long disabilities including but not limited to developmental and birth defects as well as behavioral disorders [4], [5]. The CNS is a major target for alcohol’s actions and neurological/functional abnormalities include microencephaly, reduced frontal cortex, mental retardation and attention-deficits [6]–[10]. Several mechanisms apparently contribute to the alcohol-induced disruption of fetal brain development. Among these mechanisms are suppression of protein and DNA synthesis [11], [12], inhibition of cell adhesion molecules [13] interference with cell cycle progression [14], alteration in receptor function [15]–[17], increased oxidative stress [18]–[20] altered glucose metabolism [21], [22] disruption of endoplasmic reticulum [23], altered activity of growth factors [24] or other cell-signaling pathways [25] and abberant developmental regulation of gene expression [26].

PDCD4 is a tumor suppressor, known to control critical cellular growth events predominantly by suppressing cap dependent translation via its inhibition of eukaryotic initiation factor (eIF4A) [27] and blocking of transcriptional activity of pro-survival transcription factors, AP-1 and Twist by physical interaction [28], [29]. Besides, its role in protein synthesis, PDCD4 also controls numerous genes that are implicated in cell cycle and differentiation. Studies demonstrate that PDCD4 has an inhibitory effect on cell proliferation and arrests cell cycle progression [30]. Recent studies suggest that expression of PDCD4 contributes to differentiation of skin (epidermal and hair follicles) which originates from ectoderm, which is also the origin of CNS [31]. Additionally, recent findings in *Drosophila melanogaster* germ stem cells uncovered the role for PDCD4 in stem cell maintenance and differentiation [32], [33]. Narasimhan et al., (2013) from our laboratory has demonstrated that PDCD4 is robustly expressed in rat brain cerebral cortex, cortical neurons. Importantly, developmental ethanol exposure up-regulates the expression of PDCD4 in fetal cerebral cortical neurons which mediates the inhibitory effect of the drug on protein synthesis [11]. However, the molecular mechanism underlying ethanol-induced regulation of PDCD4 is currently not clear.

Glycogen synthase kinase 3 (GSK-3) signaling pathway has been elegantly investigated with respect to embryonic brain development, regulating several downstream targets controlling diverse neural functions such as neurogenesis, neural polarization and outgrowth, synaptogenesis and neuronal migration (reviewed in [34], [35]). Activation of GSK-3β signaling in response to ETOH has been documented in cerebellar neurons [36], CNS derived PNET2 cells (primitive neuroectodermal tumor 2) [37] and neuroblastoma cells [38]. The fact that GSK-3β is imperative during neural development and that ethanol exposure modulates its activity led us to hypothesize that alcohol-induced PDCD4 is regulated via alterations of GSK-3β signaling pathway. To test this, we utilized cortical neuronal progenitors (neuroblasts) –possessing inherent characteristics of proliferation ultimately differentiating into post-mitotic neurons. Gene expression, stability, promoter based transcriptional studies showed that PDCD4 is transcriptionally upregulated by alcohol. Further using loss-of-function and pharmacological inhibition of GSK-3β, we have provided the first evidence that alcohol-enhanced PDCD4 is GSK-3β dependent. This study provides additional insights into the mechanism underlying alcohol-induced brain abnormalities occurring during early phases of fetal brain development.

## Materials and Methods

### Materials

SH-SY5Y neuroblastoma cells (CRL-2266) were purchased from ATCC (Manassas, VA). Ham’s F-12 medium, L-glutamine, actinomycin D (Act D), cycloheximide (CHX), Lithium chloride (LiCl) and anti-tubulin were purchased from Sigma- Aldrich (St. Louis, MO). GSK-3 inhibitor IV, SB −216763 was from (Millipore, Billerica, MA). Recombinant Wnt-3a was purchased from Enzo Life Sciences (Farmingdale, NY). Fetal bovine serum (FBS) (Atlanta Biologicals, Lawrenceville, GA), penicillin-streptomycin, trypsin-EDTA was purchased from (Gibco, Grand Island, NY). The antibodies used were purchased from the following companies: PDCD4 (Rockland Immunochemicals Inc., Gilbertsville, PA), phospho-p70S6Kinase, p70S6Kinase, phospho-mTOR, mTOR, phospho-GSK-3β ser 9 and phospho-GSK-3β Tyr 216 and GSK-3β, phospho-β-catenin (ser33/ser37/Thr41) (Cell Signaling Technology, Beverly, MA), β-catenin, glyceraldehyde 3-phosphate dehydrogenase (GAPDH), goat-anti-mouse IgG-HRP and goat anti-rabbit IgG-HRP (Santa Cruz Biotechnologies, Santa Cruz, CA). QuantiTect reverse transcription kit for first strand synthesis, endofree plasmid maxi kit was purchased from QIAGEN (Valencia, CA). siPORT amine was from Ambion (Austin, TX). NEB 10-beta Competent E.coli was obtained from New England BioLabs (Ipswich, MA). pGL4.16 [luc2CP/Hygro] luciferase reporter vector, pure yield plasmid miniprep system and dual-luciferase reporter 1000 assay system were from Promega (Madison, WI). PrimeSTAR Max DNA polymerase was from Takara Bio USA Inc. (Mountain View, CA). Eagle’s minimum essential medium (MEM), Trizol reagent was bought from Invitrogen (Carlsbad, CA). SMARTpool siRNA against GSK-3β was from Dharmacon Inc. (Lafayette, CO).

### Cell Culture

#### Rat brain cortical neuroblasts

We utilized spontaneously immortalized rat brain neuroblasts obtained from cerebral cortices of 18-day fetal rats (E18 neuroblasts). These cells were generously provided by Dr. Alberto Muñoz (Instituto de Investigaciones Biomédicas, CSIC, Madrid, Spain) and have been previously characterized to exhibit primitive neuronal marker nestin and NF-68 and not expresssing astrocyte marker glia fibrillary acidic protein (GFAP). They express neuron markers such as NF-145, NF-220 and neuron specific enolase after differentiation induction with dibutyryl-cAMP [39]. Cells were cultured in Ham’s F-12 media enriched with 10% FBS, L-glutamine (2 mM), streptomycin (100 µg/ml), penicillin (100 units/ml) and plasmocin (5 µg/ml). Cells were kept in an incubator maintained at 37^0^ C under an atmosphere of 95% air and 5% CO_2._ All experiments were conducted within passages 2–8.

#### SH-SY5Y culture

SH-SY5Y cells were sub-cultured using equal mixture of minimum essential medium and F-12 HAM nutrient mixture supplemented with 10% FBS, antibiotic/antimycotic and plasmocin. Cells were maintained at 37°C in a 5% CO_2_ incubator. Passages between 26–31 were used.

### Ethanol (ETOH) Treatment

Majority of the experiments were performed using ETOH concentration of 4 mg/ml (∼ 86 mM). Dose-dependent experiments were carried out using three different concentrations of 1 mg/ml (∼21 mM), 2.5 mg/ml (∼ 54 mM) and 4 mg/ml (∼ 86 mM) ETOH. To maintain ethanol concentrations in the media, we kept ETOH-treated cells in the incubator previously saturated with 100% ethanol (200 proof) and media concentration was measured using Analox AM1 alcohol analyzer (Analox Instruments, MA, USA) [11]. Control cells were maintained in ethanol-free incubator. ETOH dosage used in the study is within the physiological range and also achieved by chronic alcoholics [40].

### Cycloheximide (CHX) and Actinomycin D (Act D) Treatment

For assessment of protein stability, neuroblasts were treated with 4 mg/ml ETOH for 12 h and at the 12^th^ h CHX (20 µM) was added to inhibit protein synthesis. CHX treatment was for 1, 2 and 4 h. After treatment, cells were harvested for Western blotting analysis. To establish protein degradation (turnover) rate, the densitometrically quantified PDCD4 protein levels normalized to tubulin, were analyzed relative to levels at the beginning of CHX treatment. Likewise, for the evaluation of mRNA stability, neuroblasts were incubated with or without ETOH (4 mg/ml) for 12 h followed by Act D (1 ug/ml) treatment for 4, 8, 12 and 24 h and processed for Western and qRT-PCR analyses. No adverse effects of Act D and CHX were observed with the concentrations and time points used in the present study.

### RNA Isolation, cDNA Synthesis and Quantitative Real-time PCR (qRT-PCR)

Total RNA was extracted from neuroblasts using TRIzol reagent according to the manufacturer’s instructions (Invitrogen). 1 - 1.5 µg of total RNA was subjected to genomic DNA elimination and utilized for cDNA synthesis using Quantitect reverse transcription kit. Subsequently, cDNA samples were reverse transcribed following manufacturer’s instructions (Qiagen, Valencia, CA). For quantitative RT-PCR, 1/10^th^ of cDNA was used for amplification using predesigned Taqman gene expression assay for rat PDCD4 (Rn00573954_m1) and GAPDH (Rn01775763_g1). The cycling conditions were as follows: 50°C for 2 min, 95°C for 10 min, 95°C for 15 sec and 60°C for 1 min. Step 3–Step 4 were repeated till 39 cycles. Data was collected using CFX Manager Software and analyzed by 2^−ΔΔCt^ method to calculate relative fold change in mRNA expression.

### Western Blotting

Briefly, cells were washed in 1X PBS and lysed in radio-immunoprecipitation assay (RIPA) buffer supplemented with 1 X protease inhibitor cocktail (Sigma), sonicated (Sonics, vibra-cell ultrasonic processor) for 5 sec at an amplitude of 25% and centrifuged at 14000 rpm for 20 min at 4°C. Clarified supernatants was estimated for protein concentration and 30 µg protein was electrophoretically separated using sodium dodecyl sulfate polyacrylamide gel electrophoresis (SDS-PAGE) and electro-transferred to polyvinylidene difluoride (PVDF) membrane (Bio- Rad, CA). Non-specific binding to membrane was blocked by 5% nonfat dry milk powder in PBST. Membranes were then incubated with primary antibodies against PDCD4, phospho-p70S6Kinase, p70S6Kinase, phospho-mTOR, mTOR, phospho-GSK-3β, GSK-3β, GAPDH and Tubulin (1∶1000 or 1∶500 concentration) for 3 h or overnight. After 3 PBST washes, membranes were incubated with anti-rabbit or anti- mouse IgG secondary antibody conjugated with horseradish peroxidase (1∶10000) for 1 h. Blots were extensively washed in PBST and were developed with ECL chemiluminescence Western blot kit (Thermo scientific, IL, USA) and the signals were quantitated using Scion Image software (Scion Corporation, Frederick, Maryland, USA). The relative intensity of bands was normalized to the loading control, GAPDH or tubulin.

### Identification and Cloning of rat *Pdcd4* Promoter

The University of California, Santa Cruz (UCSC) Rat Genome Browser (Nov 2004 assembly) maps and Rat PDCD4 mRNA sequence from Genbank were used as references for the PDCD4 gene structure analysis (BC167751) (http://genome.ucsc.edu). We analyzed the genomic sequence 1046 bp upstream of the 5′ terminus of first exon of PDCD4 mRNA (BC167751) corresponding to *Rattus norvegicus* chromosome 1 assembly. 1046 bp upstream from the transcriptional start site was amplified by PCR using bacterial artificial chromosome (BAC) clone (Assembly: RGSC_v3.4; Chr: 1; Begin-End: 259983790–260217550; Library selection: CH230). PCR was performed with Prime STAR Max premix using primers containing the cushion bases followed by flanking enzymes (bold and underlined) (forward: 5′-aataat**ggtacc**gagccgtgagctgtcctagt-3′; reverse: 5′-atataa**gctagc**cgctcgctctgtttgttttt-3′). PCR was performed for 32 cycles under the following conditions: 98°C for 10 sec and 60°C for 10 sec and 72°C for 30 sec. The resulting PCR promoter fragment was purified and digested with KpnI and NheI restriction enzymes and ligated into the promoterless pGL4.16 firefly luciferase reporter plasmid to generate the PD PROM luc promoter construct. After verifying the fragment by restriction digestion and DNA sequence analysis (GENEWIZ Inc, South Plainfield, NJ), the plasmid was transformed in a NEB 10-beta competent cell E.Coli and were purified using the Plasmid Maxi Kit (Qiagen).

### Transient Transfection and Luciferase Assays

Cells were transfected using Fugene HD or XtremeGENE HP DNA transfection reagent (Roche Applied Science, IN). 200 ng/well of DNA construct (pGL4.16 or PD PROM), 3 ng of pRL-TK (Renilla luciferase for transfection efficiency), were transfected using 0.5 µl of Fugene HD or XtremeGENE HP DNA transfection reagent. Transfection was performed in Opti-MEM 1-reduced serum medium, according to the manufacturer’s protocol. 24 h post-transfection of pGL4.16 and PD PROM constructs, cells were treated with or without ETOH (4 mg/ml) for 12 h or 24 h and were lysed using reporter lysis buffer (Promega). The lysates were clarified at 14,000 rpm for 10 min and the supernatants were used for dual luciferase assay using the Dual Luciferase Reporter Assay Protocol (Promega) in Glomax 20/20 Luminometer (Promega). For analysis, Firefly luciferase enzyme activity was normalized to corresponding Renilla luciferase enzyme activity.

### Lithium Chloride (LiCl) and SB-216763 Treatment

Neuroblasts were seeded in 6 well plates at a density of 3×10^5^ cells/well. Following day, cells were treated with or without LiCl (10 mM) or SB-216763 (20 µM) for 1 h prior to 12 or 24 h ETOH (4 mg/ml) treatment. In the case of luciferase assay the inhibitor and ETOH treatment was performed 24 h post-transfection of PD PROM constructs as described above. On completion of experiments, cells were processed for downstream applications (luciferase assay, Western and quantitative real time RT-PCR).

### Small Interfering RNA (siRNA) Transfection

Cells were seeded in 6 well plate at a density of 3×10^5^ cells/well. Following day, cells were transfected with either non-targeting siRNA (scr siRNA, 100 nM) or siRNA targeting GSK-3β (si GSK-3β, 100 nM). Prior to transfection, cells were replaced with 800 µl of fresh media and transfected with 200 µl of transfection complex. 24 h later, cells were treated with or without 4 mg/ml ETOH for additional 24 h and processed for either Western blotting or RT-PCR analysis.

For experiments involving siRNA followed by luciferase assays, cells were seeded in 12 well plates at a density of 1.5×10^5^ cells/well. Cells were pre-transfected with scr siRNA or si GSK-3β for 24 h and followed by reporter construct transfection. Post-transfection of the constructs, cells were exposed to ETOH (4 mg/ml) for 24 h. Lysates were then processed for dual luciferase assays as described above.

### Statistical Analysis

All results are expressed as mean ± SEM. For comparing more than two groups, one way analysis of variance (ANOVA) followed by Student–Newman–Keul’s post hoc analysis was used to determine statistical significance. For some experiments two way analysis of variance followed by Bonferroni post hoc tests were used. Student’s *t*-test was used for experiments involving only two groups. p<0.05 was considered as statistically significant. All statistical analysis was conducted using GraphPad Prism software. The “*n*” number given in the figure legend is common to individual panels within a figure.

## Results

### Ethanol Induces PDCD4 Protein Expression in Cortical Neuroblasts

**Figure 1:**
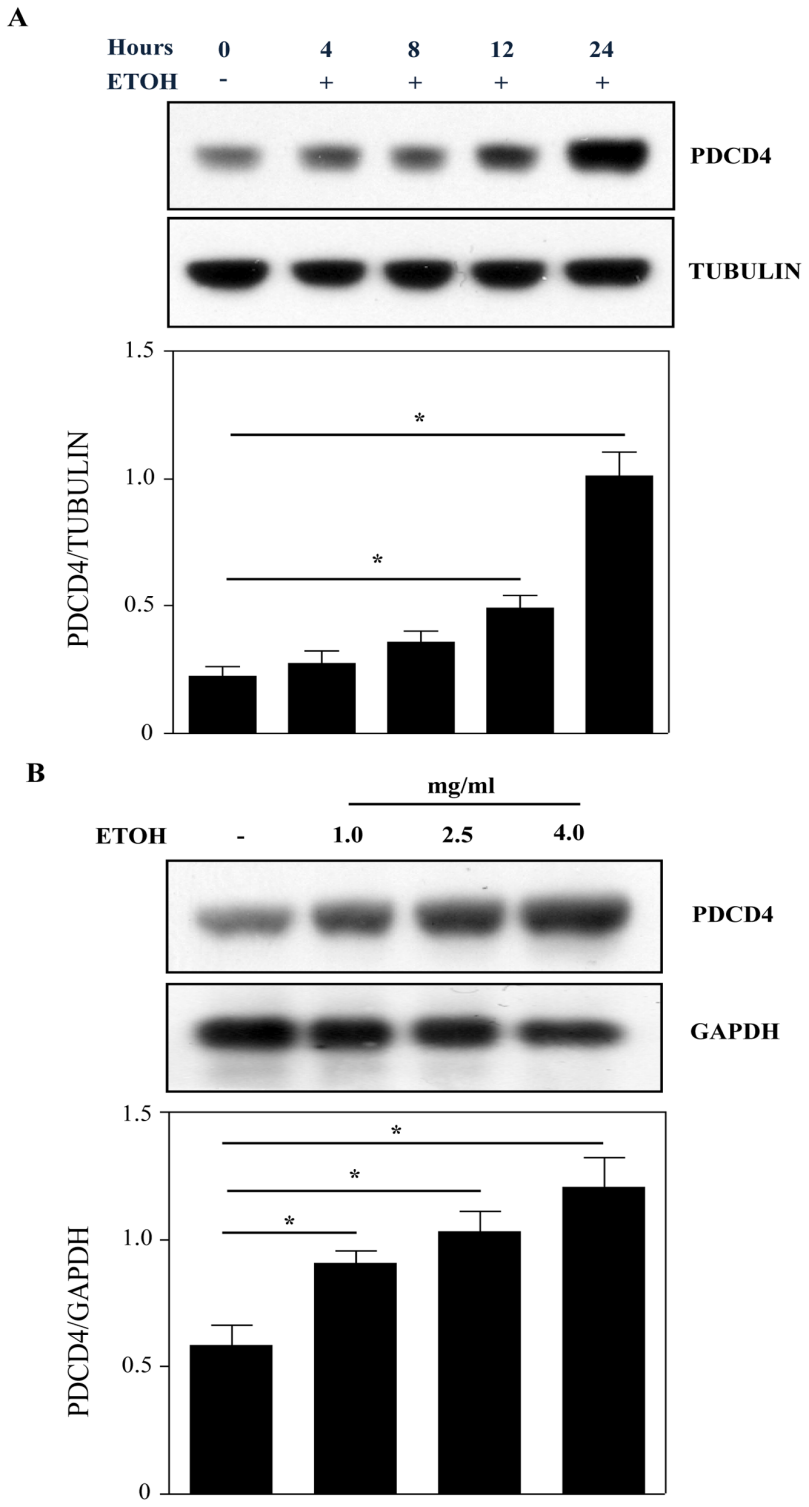
Ethanol time and dose dependently increases PDCD4 expression in cortical neuroblasts. (**A**) Cells were treated with or without ETOH (4 mg/ml) for 4, 8, 12 and 24 h. PDCD4 levels in neuroblasts were evaluated by immunoblot analysis. Anti-tubulin was used to demonstrate equal loading. Lower panel shows densitometric scanning analysis ratio of PDCD4 to tubulin. (**B**) Cells were treated with 1 mg/ml (∼21 mM), 2.5 mg/ml (∼54 mM) and 4 mg/ml (∼86 mM) ethanol for 24 h. Cells were then harvested and immunoblotted with anti-PDCD4 and anti-GAPDH. Protein levels were quantitated using Scion Image software. One-way analysis of variance (ANOVA) and Newman-Keul’s posthoc test was performed to establish statistical significance.*p<0.05 when compared with untreated control (A & B), n = 3.

Neuron generation during brain development involves neuroblast lineage progression that encompasses assymetrical division of neural progenitors, cell cycle exit and differentiation [41], [42]. Our laboratory has previously shown that PDCD4 plays a critical role in ethanol-induced dysregulation of protein synthesis in PCNs and *in utero* binge alcohol model [11]. Since neuron development is progeny dependent, in the current study we investigated whether ethanol-induced PDCD4 changes are conserved in mitotic neuroblasts, the immediate precursor of neurons. This would facilitate understanding to what extent and how early the damage to the developing brain is inflicted by ethanol. We used spontaneously immortalized rat brain neuroblasts, an established *in vitro* model to study developmental brain signaling events [43]. We utilized a range of ETOH concentrations (21 mM to 86 mM) that has been demonstrated to produce *in vitro* changes that are comparable to alcohol-exposed animals with blood alcohol levels of ∼150 mg/dl and also achieved by binge alcohol consumption [44]. Treatment of neuroblasts with 4 mg/ml of ETOH for different periods resulted in increased PDCD4 protein levels. ETOH treatment resulted in a statistically significant (p<0.05) increase by ∼2.0 and ∼4.0 fold at 12 and 24 h respectively (Figure 1A). We next used three different concentrations of ETOH (1.0, 2.5 and 4 mg/ml) to determine the dose dependent effects on PDCD4 protein expression. Figure 1B demonstrates that ETOH treatment dose dependently increased PDCD4 protein expression in neuroblasts (p<0.05 *vs*. control, compare lanes - 2,3,4 *vs* 1). These results indicates that ETOH time and dose-dependently upregulates PDCD4 protein in proliferating neuroblasts, similar to our prior findings in postmitotic neurons. This further suggests that in developmental ethanol toxicity, PDCD4 could play a critical role and its regulation is likely conserved.

### Ethanol Up-regulation of PDCD4 Expression is not caused by Increased Protein Stability

**Figure 2:**
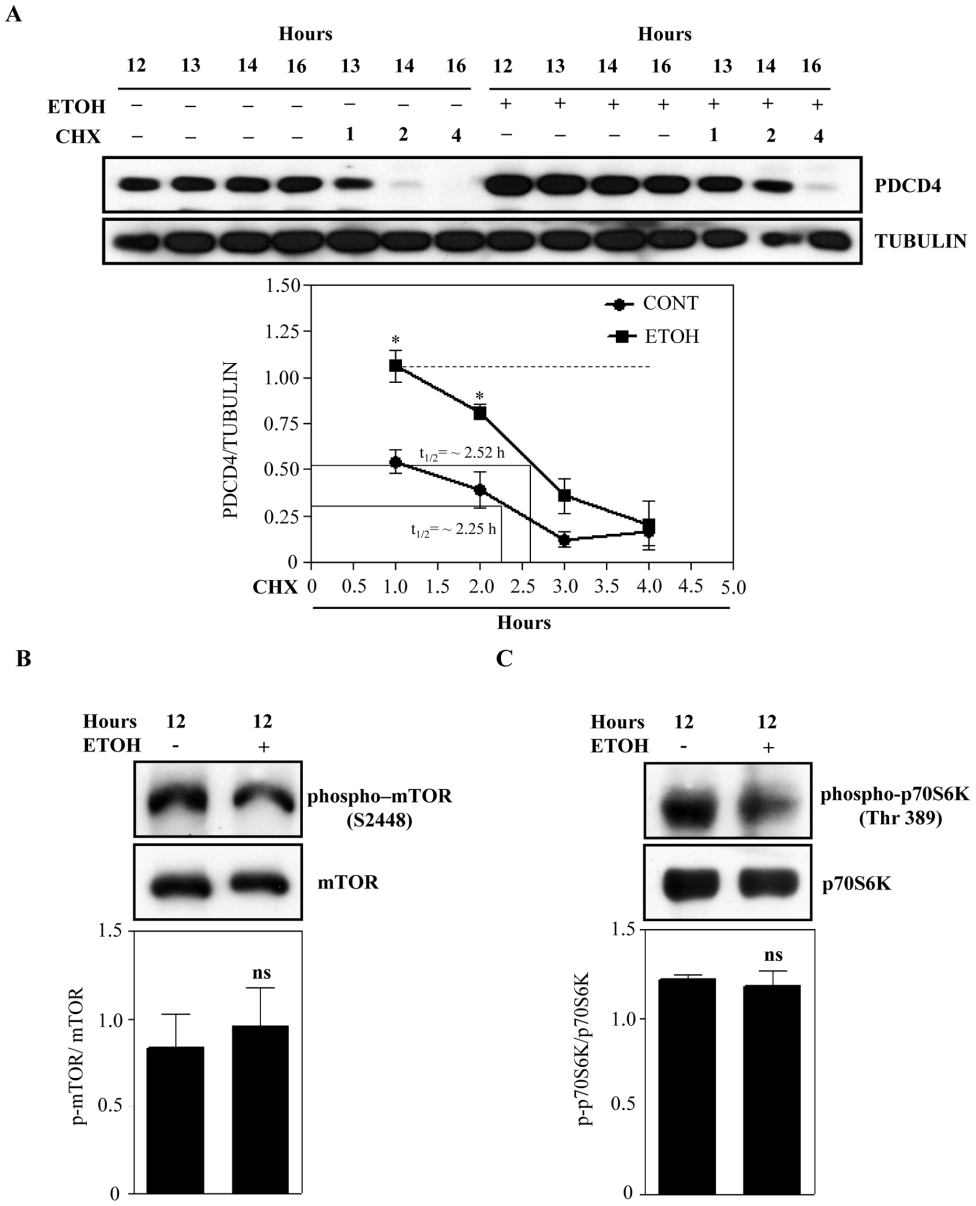
Ethanol does not stabilize PDCD4 protein. (**A**) Neuroblasts were incubated with or without ETOH (4 mg/ml) for the times indicated followed by CHX (20 µM) treatment for 1–4 h. Total cell lysates were resolved by SDS-PAGE and immunoblotted with antibodies against PDCD4 or tubulin (top panel). The signals were quantitated by densitometry and the ratios of PDCD4 over tubulin were plotted (bottom panel). Two-way analysis of variance (ANOVA) with Bonferroni post hoc tests was carried out to establish statistical significance.*p<0.05 when compared with untreated control. (**B**) Top panel represents the immunoblot analysis against phosphorylated form of mTOR (S2448) and mTOR from untreated and ETOH treated cell lysates. The signals for the bands were quantitated densitometrically and the intensity of phospho-mTOR relative to the levels of mTOR protein expression was calculated (ns not significant when compared to control as determined by student’s t-test) (lower panel). (**C**) Western blot analysis of phospho-p70S6Kinase (Thr 389) and p70S6Kinase on untreated and ETOH treated whole cell lysates (top panel). Bottom panel illustrate the densitometric quantification of phospho-p70S6Kinase to total p70S6Kinase (ns, not significant compared with untreated control as analyzed by student’s t-test). n = 3.

The two major events that could control gene expression are transcription (mRNA synthesis) [45], [46] and translation (protein synthesis) [47]. Post-transcriptional mechanisms involving decreased mRNA degradation or increased mRNA stability and those involving decreased protein degradation or increased protein stability, could also influence gene expression [48], [49]. Therefore, we examined how PDCD4 could be regulated by ethanol in cortical neuroblasts. mTOR/p70S6Kinase-mediated phosphorylation of PDCD4 results in degradation of PDCD4 by the ubiquitin ligase βTRCP [50]. Since PDCD4 protein is a target for degradation, we first investigated whether ETOH-induced increase in PDCD4 protein levels is due to protein stability using cycloheximide (CHX) experiments. Densitometric quantification of Western blotting analysis showed a significant increase in the expression of PDCD4 with ETOH treatment compared to control (p<0.05, compare lanes 8–11 *vs* lanes 1–4; Figure 2 A). However, a delayed trend was noted in the rate of PDCD4 decay (ETOH+CHX- t1/2 ∼ 2.52 h *vs* untreated+CHX- ∼2.25 h; t1/2 in Figure 2A, lower panel) following the addition of CHX. To further confirm that stability based mechanisms are not primarily involved in PDCD4 upregulation by ETOH, immunoblotting analysis for activation of mTOR and p70S6Kinase was performed using phosphorylation specific antibodies for mTOR (S2448) and p70S6Kinase (Thr389) and we observed no statistical changes in their phosphorylation (Figure 2B and 2C). This excludes a role for the mTOR/p70S6K pathway in controlling ETOH-induced PDCD4 regulation. If stability based control were in play, one would have expected the increased PDCD4 protein to be maintained in the presence of CHX+ETOH (indicated by dotted line). Altogether, these data suggests that ETOH mediated PDCD4 protein increase is not due to PDCD4 protein stability (Figure 2A, B and C).

### Ethanol Induces PDCD4 mRNA and does not Affect mRNA Stability

**Figure 3:**
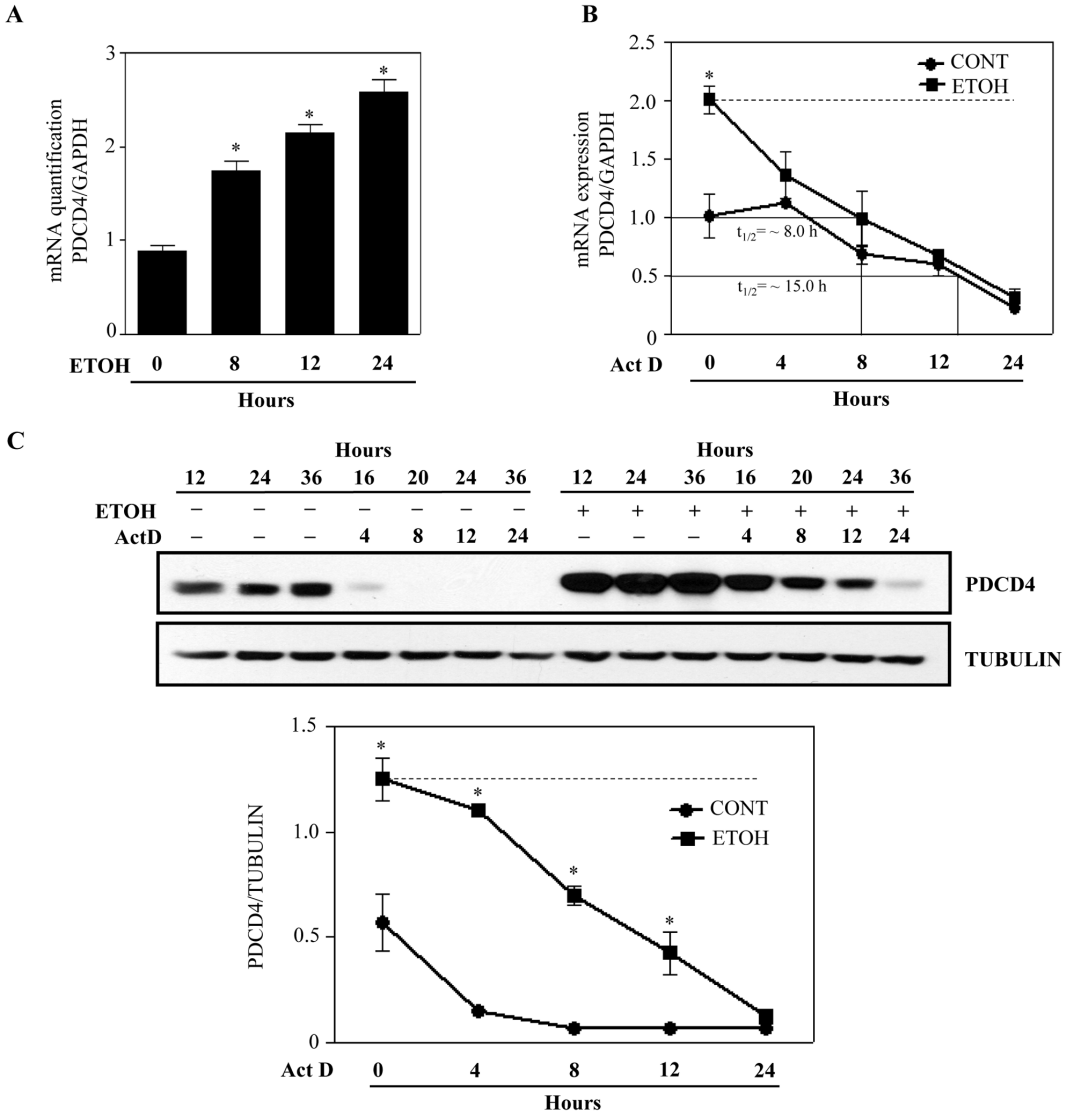
Ethanol exposure induces PDCD4 independent of mRNA stability in neuroblasts. (**A**) Quantitative real time PCR was performed on mRNA obtained from neuroblasts treated with or without ETOH (4 mg/ml) for indicated time periods. *Pdcd4* expression was determined after normalizing to GAPDH mRNA expression. Statistical significance was established by one-way ANOVA and Newman-Keul’s posthoc test.*p<0.05 when compared to control. (**B & C**) Cells were treated with or without 4 mg/ml of ETOH followed by Act D (1 µg/ul) treatment for indicated time periods (4, 8, 12 and 24 h) and cells were harvested at the end of the experiments for either PDCD4 or GAPDH mRNA analysis by qRT-PCR (**B**) or protein analysis by Western blotting (**C**). Statistical analysis was carried out by two-way analysis of variance (ANOVA) with Bonferroni post hoc tests.*p<0.05 compared with control. n = 3.

In view of the fact that PDCD4 is regulated by miR-21 [51], [52], we next determined if ethanol regulates PDCD4 at the post-transcriptional level. To assess this, we first tested whether ETOH induces PDCD4 message using real time PCR analysis. ETOH time dependently effected a significant (p<0.05) increase in PDCD4 mRNA (Figure 3A). The effect was observed only beyond 4 h of ETOH treatment (4 h data not shown). To further elucidate whether ETOH-dependent increase in PDCD4 mRNA levels is due to an increased half-life of the transcripts, mRNA stability experiments were performed using Act D to arrest *de novo* mRNA synthesis. Real time-PCR analysis demonstrated that PDCD4 mRNA levels decreased with Act D treatment irrespective of ETOH exposure in neuroblasts (Figure 3B). This suggests that ETOH mediated PDCD4 mRNA increase is not due to PDCD4 mRNA stability. Further analysis of the Act D exposed samples for PDCD4 protein expression revealed a delayed decay in response to ETOH (∼80% decay in control, 4 h *vs* ∼15% in ETOH, 4 h) (Figure 3C). This delay in PDCD4 protein decay appears to be maintained until 12 h (control ∼95% *vs* ∼60%). Interestingly, this trend is seen when PDCD4 mRNA is decreased pointing out a compensation based sustenance of PDCD4 protein (when mRNA is blocked) during combined stress (ACT D+ETOH) (Figure 3C). These results also highlight regardless of co-existence of any other cellular stress along with ETOH (Act D/CHX), PDCD4 changes are sensitive to ETOH. To note, Lu et al. [52] has reported that miR-21 translationally represses PDCD4 as against the normal post-transcriptional based silencing. At this point, though the mechanism is not clear, we speculate that during a global inhibition of *de novo* mRNA synthesis using Act D, miR-21 biogenesis could also be affected. Given these facts, in this context, we hypothesize an Act-D induced miR-21 reduction might relieve the translation check that it had on PDCD4. Overall, these data suggests that ETOH-induced PDCD4 changes are not dependent on mRNA stability.

### Ethanol Transcriptionally Activates PDCD4 Expression in Cortical Neuroblasts

**Figure 4:**
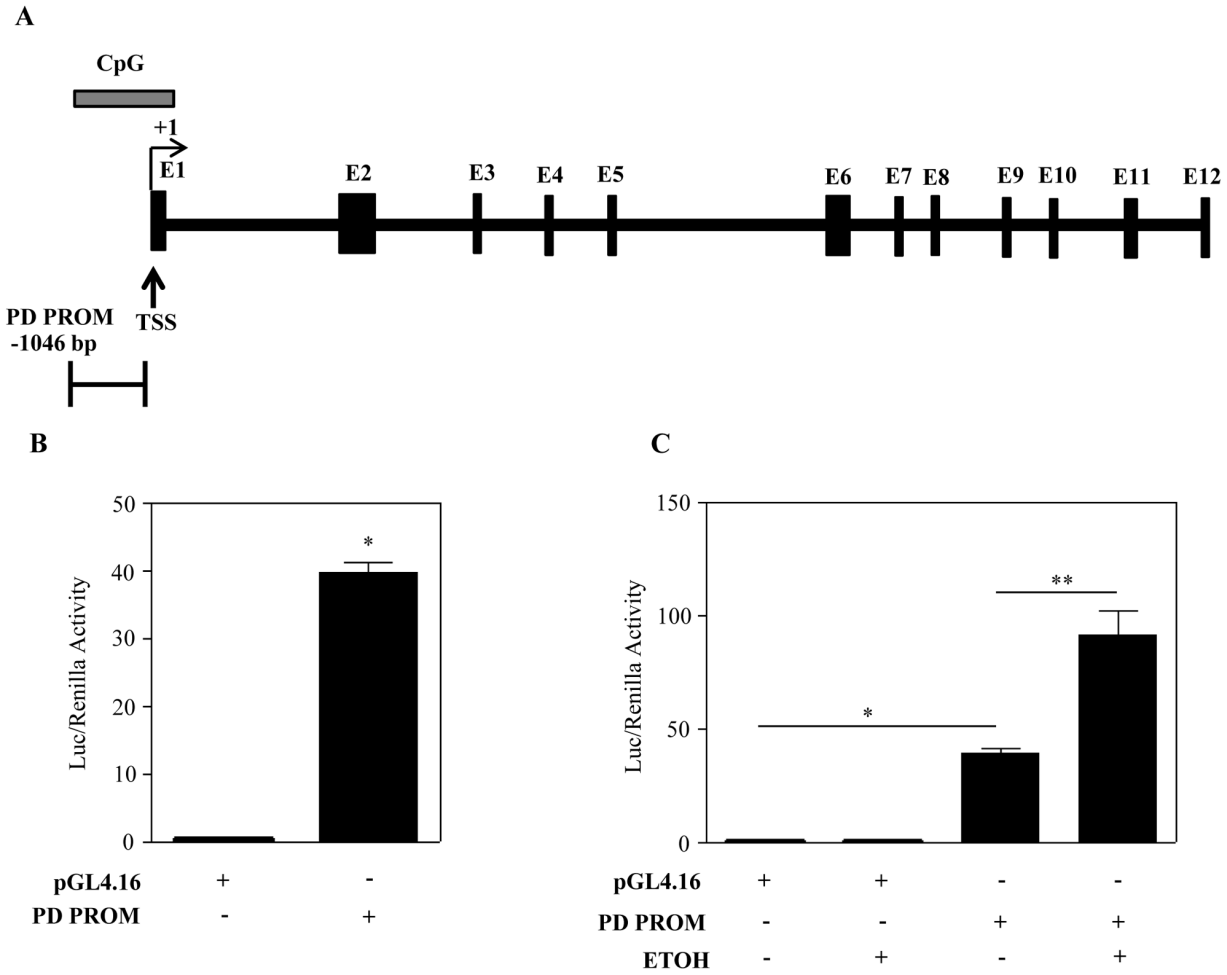
Ethanol transcriptionally upregulates *Pdcd4*. (**A**) Rat *Pdcd4* gene structure derived from GenBank (Accession No. BC167751). *Pdcd4* consists of 12 exons (E1–E12) out of which E2–E12 are coding exons and E1 is a non-coding exon which forms the 5′ untranslated region. Sequences 1046 bp upstream of the transcriptional start site (TSS) regarded as *Pdcd4* promoter was used in the present study. The transcriptional start site is designated as +1 and CpG island is represented by the horizontal bar above E1 of rat *Pdcd4* gene. (**B**) Cells were transfected with either pGL4.16 or PD PROM constructs and luciferase activity was evaluated 24 h post-transfection using dual luciferase reporter assay system. Data was analyzed using Student’s t-test. *p<0.001. (**C**) Neuroblasts were transfected with either pGL4.16, promoterless or PD PROM (−1046) for 24 h. Following transfection, cells were treated with or without 4 mg/ml of ETOH for 12 h and were processed for luciferase activity. Data was analyzed using one-way ANOVA and Newman-Keul’s posthoc test.*p<0.001. n = 6.

We have shown that ETOH induced PDCD4 transcript is not influenced by mRNA stability which suggests that the regulation could be at the transcriptional level. As described above in the experimental section, UCSC genome browser and Genbank (Accession No. BC167751) were used as references for the prediction of putative *Pdcd4* promoter. The rat *Pdcd4* gene is located on chromosome assembly 1q55 and contains 12 exons (E1–E12) where E1 is a non-coding exon which forms the 5′ untranslated region (Figure 4A) and so far, two splice variants of rat *Pdcd4* is known [53]. The 5′-most nucleotide of the largest cDNA clone available (Genbank accession # BC167751.1) was designated as transcriptional start site. Analysis of this region indicated the presence of a CpG island which is represented by the horizontal bar above E1 of rat *Pdcd4 gene*. In general, CpG islands are typically found near transcription start sites (TSS, Fig. 4A), and are considered to be one of the most reliable predictors of promoter in the mammalian genome other than TATA box and initiator region (Inr) [54]. Subsequently, the putative promoter chosen by us for this study was also validated using two bioinformatics based tool provided by RIKEN and http://rulai.cshl.edu (Accession No.86712). A 1046 bp segment upstream of the 5′ flanking region of E1 of *Pdcd4* gene representing putative rat *Pdcd4* promoter (PD PROM) was PCR amplified using BAC clone (Figure S1) which was subsequently cloned into a reporter construct and sequence verified. Transient transfection of PD PROM demonstrated increased luciferase activity compared to pGL4.16 (Figure 4B) confirming that indeed the genomic fragment exhibited transcriptional activity. Next, we examined the effect of ETOH on PD PROM activity. As shown in Figure 4C, PD PROM reporter activity was significantly increased (p<0.001) by ∼ 2- fold when exposed to ETOH. These results indicate that ETOH induced *Pdcd4* expression occurs at the level of gene transcription.

### Ethanol Activates GSK-3β in Cortical Neuroblasts

**Figure 5:**
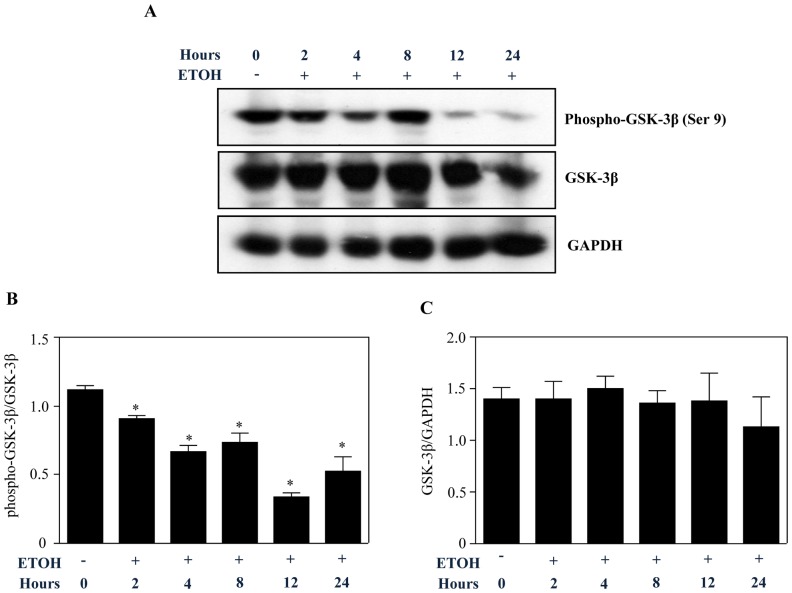
Ethanol activates GSK-3β. Neuroblasts were treated with ETOH for 2, 4, 8, 12 and 24 h. Protein levels of phospho-GSK-3β (Ser 9) were determined in control and ETOH treated cells by Western blot analysis (top). Statistical significance was evaluated by normalizing with GSK-3β and GAPDH (bottom). Analysis was performed using one-way ANOVA followed by Newman-Keul’s posthoc test. *denotes p<0.05 compared with control. n = 3.

Having established that ETOH transcriptionally activates PDCD4, we next explored for the possible regulator involved in this control. Recent studies have documented that ETOH promotes glycogen synthase kinase 3β (GSK-3β) signaling in CNS and modifies critical neurogenetic processes by regulating downstream targets. Therefore, we tested whether ETOH’s induction of PDCD4 is mediated by GSK-3β activation. To test this assertion, we performed Western blotting on untreated and ETOH-treated cell lysates to determine GSK-3β kinase phosphorylation. Phosphorylation at Ser 9 negatively regulates the activity of GSK-3β whereas phosphorylation at Tyr 216 positively regulates its activity [55]. Using GSK-3β Ser 9 specific phospho antibody, we demonstrated that ETOH-treatment significantly decreased the inhibitory phosphorylation starting from 2 to 24 h compared with the control (Figure 5A and 5B). The later time points demonstrated a remarkable reduction in Ser 9 phopshorylation indicating enhanced activity of GSK-3β (compare lanes 5, 6 *vs* 1; Figure 5B). While no changes in GAPDH normalized total GSK-3β levels were observed (Figure 5C). In addition, Tyr 216 phosphorylation of GSK-3β was found to be unchanged in response to ETOH treatment (Figure S2). As a GSK-3β functional assay, phosphorylation of one of its substrates, β-catenin at Ser33/Ser37/Thr41, was assessed using phospho-specific (Ser33/Ser37/Thr41) antibody. Phosphorylation at these sites by GSK-3β destabilizes and degrades β-catenin [56]. Evidently we observe a significant decrease in β-catenin protein expression on alcohol treatment (Figure S3). This was paralleled by an increase in GSK-3β specific phosphorylation of β-catenin at Ser33/Ser37/Thr41 (Figure S3). This suggests that Tyr 216 phosphorylation does not contribute to the activity of GSK-3β and in fact, the decrease in ser 9 inhibitory phosphorylation (Fig. 5) is sufficient to keep GSK-3β active. It has been suggested that activation of GSK-3β could occur independent of changes observed in Tyr 216 or Ser 9 involving several post-translational mechanisms [57], [58].

As Wnt-3a is a negative regulator of GSK-3β, we next assessed the role for Wnt-3a in GSK-3β-mediated PDCD4 regulation utilizing recombinant Wnt-3a experiments. We noted that Wnt-3a treatment decreased resting PDCD4 expression suggesting a Wnt3a/GSK-3 signaling in PDCD4 regulation under basal conditions (lane 1 *vs* 3; Figure S4). Further, Wnt-3a pretreatment significantly decreased ETOH-induced PDCD4 protein expression (lane 2 *vs* 4; Figure S4). In support to this finding, Vangipuram and Lyman (2012) have documented that ethanol has a negative impact on Wnt/GSK-3β/β-catenin signaling pathway in human neural stem cells [59]. Our future study will address as to how GSK-3/catenin signaling downstream of Wnt-3 regulates PDCD4 expression. Altogether, these results suggest that Wnt-3/GSK-3β/catenin pathway might control PDCD4 regulation and that a decrease in GSK-3β ser9 phosphorylation status along with unknown post-translational modifications might have a predominant influence on activating GSK-3β in response to ETOH in neuroblasts.

### Pharmacological Inhibition of GSK-3β Blocks ETOH-induced PDCD4

**Figure 6:**
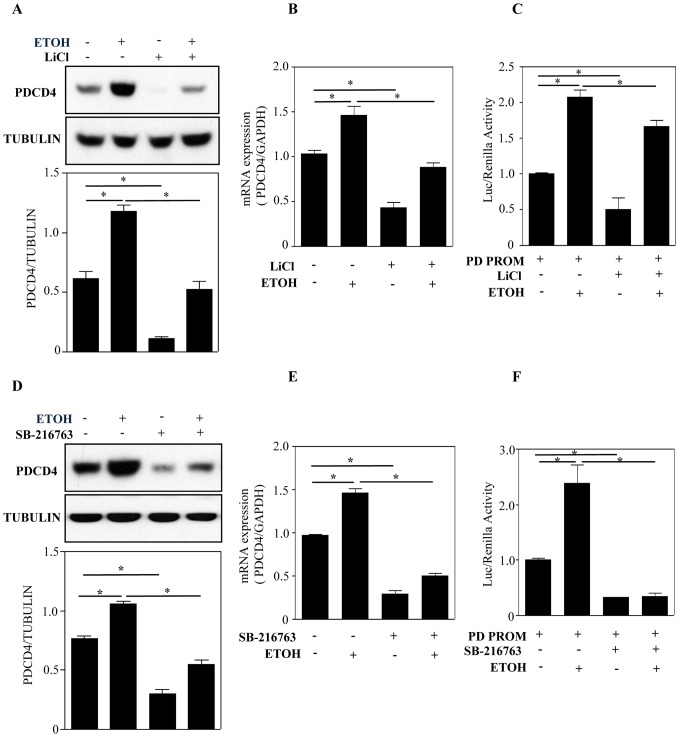
Chemical inhibition of GSK-3β signaling pathway increases PDCD4 expression. Cells were pre-treated with with LiCl (10 mM) or SB-216763 (20 µM) for 1 h and then exposed with ETOH for 12 h. Panel A shows Western blot and densitometric scanning analysis of PDCD4 and tubulin with LiCl and ETOH treatment. (**B**) At the end of LiCl and ETOH treatment, cells were processed for qRT-PCR analysis to determine PDCD4 mRNA expression. Results are expressed as fold change in mRNA relative to GAPDH control. (**C**) Cells were transfected with either promoterless, pGL4.16 plasmid or 1046 bp PD PROM construct for 24 h. Following transfection, cells were pretreated with LiCl for 1 h and then exposed with ETOH for 12 h. After treatment, cells were processed for determining luciferase activity. Data are expressed as fold change relative to control. **D**, **E** and **F** depicts the Western blot analysis, RT-PCR analysis (expressed in fold change) and relative luciferase activity (expressed in fold change) when treated with SB-216763, a known GSK-3β inhibitor. In A–F, statistical analysis was performed using one-way ANOVA followed by Newman-Keul’s posthoc correction. *is statistically significant at p<0.05. n = 6.

So far we have shown that ETOH upregulates PDCD4 beyond 8 h at the same time activating GSK-3β as early as 2 h. Therefore, we next tested whether this GSK-3β activation regulates PDCD4 using pharmacological inhibitors, lithium chloride (LiCl) and SB-216763. GSK-3β activity was evaluated using an antibody specific for inhibitory phosphorylation at Ser-9 which was observed to be enhanced on chemical inhibition using LiCl (Figure S5). Cells treated with LiCl or SB-216763 showed a significant decrease in basal PDCD4 protein expression (p<0.05 *vs*. control) (lane 1 *vs* lane 3; Figure 6A & D). LiCl or SB-216763 pretreatment blocked ETOH-induced PDCD4 protein expression significantly (p<0.05) (lane 2 *vs* lane 4; Figure 6A & D) suggesting a role for GSK-3β in ETOH-specific regulation of PDCD4 protein. Similarly, the mRNA levels of PDCD4 induced by ETOH was significantly blocked with LiCl or SB-216763 pretreatment (p<0.05) (lane 2 *vs* lane 4; Figure 6B & E). This suggests that changes in GSK-3β dependent PDCD4 protein is mediated through PDCD4 mRNA alterations. Further analysis of PD PROM activity demonstrated a significant decrease in the transcriptional activity in cells that were pre-treated with LiCl or SB-216763 compared to controls (lane 1 *vs* lane 2; Figure 6C & F). Moreover, ETOH-induced PD PROM activity was also significantly (p<0.05) blocked by the GSK-3β inhibitors (lane 3 *vs* lane 4; Figure 6C & F). Altogether these data suggests that ETOH-induced PDCD4 upregulation is under the control of GSK-3β. Since we did not observe a total blockade of PDCD4 expression by GSK-3β inhibition, we do not exclude the interplay of other mechanisms in regulating PDCD4.

### siRNA Mediated Silencing of GSK-3β Regulates PDCD4

**Figure 7:**
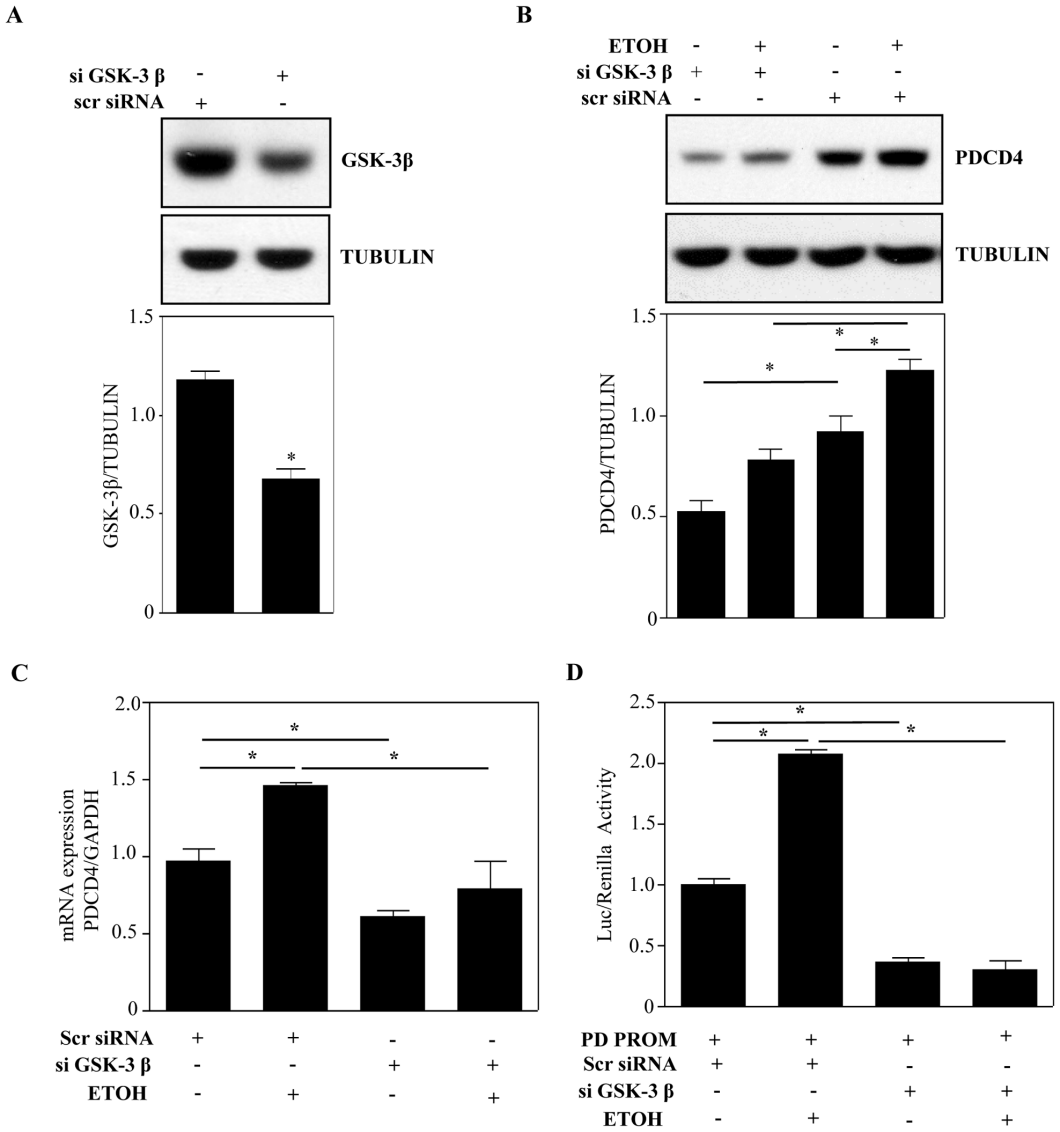
Downregulation of GSK-3β blocks basal as well as ETOH-induced PDCD4 expression. (**A**) Cells were transfected with 100 nM siRNA against GSK-3β (si GSK-3β) or 100 nM of scramble siRNA (scr siRNA). 24 h post-transfection cell lysates were immunoblotted with anti-GSK-3β and tubulin. Lower panel shows densitometric scanning of GSK-3β normalized to tubulin. (**B**) Western analysis of PDCD4 on cell lysates transfected with si GSK-3β (100 nM) followed by ETOH treatment for 24 h (top panel). PDCD4 and tubulin specific signals were measured by densitometry and the ratios of PDCD4 over tubulin were plotted (bottom panel). *p<0.05 shows statistical significance using one-way ANOVA following Newman-Keuls posthoc test. (**C**) Quantitative q-RT PCR analysis for PDCD4 transcript expression. Data is expressed as fold change relative to control. ANOVA was performed and *indicate statistical significance at p<0.05 (**D**) 24 h post-transfection of respective siRNAs (as in Panel A), pGL4.16 or PD PROM luc contructs were transfected. 24 h after transfection cells were treated with or without ETOH for additional 24 h and cell lysates were subjected for dual luciferase assay. Results are expressed as fold change relative to control and data analyzed by ANOVA and Newman-Keul’s posthoc test. *denotes p<0.05. n = 3.

Specificity concerns in usage of pharmacological inhibitors led us to use gene silencing based strategy to elucidate the molecular involvement of GSK-3β in ETOH-induced PDCD4 regulation. First, the efficiency of GSK-3β specific siRNA in knocking down GSK-3β expression was tested using immunoblotting. A clear decrease in GSK-3β levels by ∼50% was observed in cells transfected with GSK-3β siRNA when compared to scrambled nontargeting siRNA (Figure 7A). Using this loss-of-function strategy, we next demonstrated that GSK-3β siRNA transfection by itself significantly decreased the expression of PDCD4 (lane 3 *vs* lane 1; Figure 7B) (p<0.05) suggesting a role for GSK-3β in basal PDCD4 expression. Though a significant downregulation of PDCD4 is achieved by blockade of GSK-3β, a notable residual level of PDCD4 is still observed which may be attributed by incomplete GSK-3β silencing (lane 2 *vs* lane 1; Figure 7A). Further, ETOH-induced PDCD4 was also significantly (p<0.05) blocked by the downregulation of GSK-3β (Figure 7B; lane 4 *vs* lane 2). In a similar manner, downregulation of GSK-3β resulted in a significant reduction of both basal (lane 1 *vs* lane 3; Figure 7C) as well as ETOH-induced PDCD4 mRNA levels (p<0.05) (lane 2 *vs* lane 4; Figure 7C). Additionally, we determined whether the above GSK-3β dependent changes in *Pdcd4* message is influenced at the level of gene transcription using promoter assays. Figure 7D shows a significant decrease in PD PROM luciferase activity in cells transfected with GSK-3β siRNA when compared to scrambled siRNA (p<0.05) (lane 1 *vs* lane 3). Over and above ETOH-induced PD PROM activity was remarkably blocked in GSK-3β silenced cells (lane 2 *vs* lane 4; Figure 7D). Altogether, these data strongly indicate a molecular involvement of GSK-3β in regulating *Pdcd4* gene expression under resting as well as ETOH-inducible state in cortical neuroblasts.

## Discussion

Consumption of alcohol during the sensitive periods of neurogenesis can lead to developmental brain disabilities associated with FAS [60]. In post-mitotic neurons and in cerebral cortex of a rat FAS model, we previously reported a novel molecular mechanism involving PDCD4 by which ETOH suppresses protein synthesis, a process that is critical for brain development. PDCD4 endowed with a capability of suppressing translation is known to play a pivotal role in cell proliferation, differentiation, migration, invasion, inflammation, apoptosis, drug sensitivity, tumorigenesis and progression [61]. Alcohol has been shown to affect essential processes such as neuronal migration, neurogenesis and gliogenesis during the early phases of brain development [62]. Given this premise, along with scant information, it is essential to uncover the potential mechanisms underlying PDCD4 regulation during ethanol-neurotoxicity. To this end, we identified for the first time, a role for GSK-3β in the transcriptional control of PDCD4 during normal as well as ETOH-stressed neurogenetic processes using immediate neuronal precursor cellular system.

Our current study reinforces the notion that ETOH disrupts PDCD4 expression in neuroblasts (Figure 1) akin to our previous findings in primary cortical neurons suggesting that PDCD4 could be alcohol-sensitive and a developmentally regulated gene in brain. Therefore, any distortion in the PDCD4 regulatory network during the vulnerable period of cortex development by ETOH is expected to strongly impact fetal cortical architecture. Our study provides a crucial link between ethanol and a critical molecule that is involved in cellular differentiation. PDCD4 has been widely reported to be regulated post-translationally by mTOR/p70S6kinase/β-TRCP dependent proteasomal degradation and post-transcriptionally by miR-21 [50], [63]. However, the current study using CHX and ACT D excludes the role for above mechanisms in ETOH-induced upregulation of PDCD4 in neuroblasts (Figure 2 & 3). On the contrary, we observe increase in PDCD4 levels even when the existing transcripts are rapidly eliminated (ETOH+ACT D - t1/2 ∼ 8h *vs* untreated+ACT D - t1/2 ∼15h). These observations explain the existence of inductive phenomenon for ETOH-specific PDCD4 regulation in addition to supporting the claim that transcripts with faster decay undergo larger induction [64]. Such a relationship has already been documented as a fundamental principle for a category of mRNAs involved in transcription, signal transduction and stress-response [64], [65].

Transcriptional induction is the first level of gene regulatory control. In line with this, our studies with PDCD4 promoter demonstrated that ETOH transcriptionally upregulates PDCD4 gene expression in brain neuroblasts (Figure 4) and points out involvement for the ∼1 kb proximal promoter fragment upstream of transcription start site. PDCD4 has been already reported to be transcriptionally controlled by v-myb, Sp1, ZBP-89, Smad3, RAR-α [66]–[69]) in non-neuronal systems. Our experiment with neuroblasts point out that GSK-3β specific phosphorylation of β-catenin is increased and subsequently, the expression of β-catenin is reduced in response to ETOH treatment (Figure S3). It has been reported that Wnt/β-catenin signaling is appropriately regulated by the protein level of β-catenin that in turn is modulated by its phosphorylation [70]. This suggests that the decrease in β-catenin level dependent on its phosphorylation could likely derepress the effect that it had on Pdcd4 promoter resulting in an increase in *Pdcd4* expression. Several studies have shown that β-catenin could repress genes such as Tcf3, NFκB, 15-PGDH which is derepressed and activated upon knockdown or transcriptional inactivation of β-catenin [71]–[73]. Gleaned from these studies and our data, we speculate that PDCD4 could be yet another derepressed target of β-catenin signaling which is under investigation.

In general, stimuli from extracellular milieu are conveyed via signaling intermediates involving several protein kinases to regulate gene expression. In this context, exploring for possible regulators in the form of stress kinases led to the discovery of GSK-3β in controlling ethanol induced regulation of PDCD4 in neuroblasts. Interestingly, pharmacological inhibition and molecular loss-of-function approaches showed a role for GSK-3β in baseline regulation of PDCD4 in rat cortical neuroblasts. GSK-3β dependent basal regulation of PDCD4 was also noted in human dopaminergic SH-SY5Y neuronal cell model signifying the conservancy of PDCD4 regulation among different species (rat and human) along with developmental stages of neuronal lineage (neuro-precursor mitotic neuroblasts and differentiated neurons) (Figure S6 & Figure S7). GSK-3β is a key constituent of canonical Wnt/β-catenin signaling pathway in vertebrates and wingless signaling pathway in Drosophila and is long known to play significant role in embryonic brain development (reviewed in [74], [75]). Alterations in GSK-3β activity produces abnormalities in neurogenesis, synaptogenesis, cell polarity, neuronal migration, axon growth, neuronal plasticity, which has also been pertinent with ethanol insult (reviewed in [34], [35], [76], [77]. Though a positive role for GSK-3β involvement in PDCD4 protein regulation has been recently reported in lung cancer cells [78], our studies provide the first evidence for transcriptional regulation of PDCD4 by GSK-3β in a developmental neuronal setting. Furthermore, our results point to an abnormal expression of PDCD4 induced by ETOH via GSK-3β signaling pathway may underlie neurogenetic abnormalities seen with FAS. Of note, the importance of PDCD4 in brain cell proliferation, maintenance or differentiation is been investigated in our laboratory. Taken together, we propose that ablation of GSK-3β/PDCD4 network and/or identification of vital regulatory motifs of the PDCD4 promoter that are responsive to ETOH will enable generation of better molecular checkpoints in mitigating neurotoxic effects of ETOH during development.

## Supporting Information

Figure S1 **Amplification of −1046 bp rat** ***Pdcd4*** **promoter fragment.** Agarose gel electrophoresis showing the amplification of putative *Pdcd4* promoter fragment of 1046 (PD PROM) using the primers described in methods section. PCR product was resolved in 1% agarose gel and visualized by staining with ethidium bromide. Lane 1 and lane 2 depicts 1 kb ladder and PCR amplification product respectively.
(TIF)

Figure S2 **Effect of ethanol on GSK-3β Tyr 216 phosphorylation.** Neuroblasts were treated with ETOH (4 mg/ml) for 2, 4, 8, 12 and 24 h. Tyrosine phosphorylation of GSK-3β was determined in control and ETOH treated cells by Western blot analysis using p-GSK3Tyr 216 specific antibody (top). Statistical significance was evaluated by normalizing with GSK-3β and tubulin (bottom). Statistical analysis was performed using one-way ANOVA followed by Newman-Keul’s posthoc test. Datapoints were not significant when compared to untreated control, n = 3.
(TIF)

Figure S3 **Ethanol enhances phosphorylation and degradation of β-catenin.** Neuroblasts were treated with ETOH (4 mg/ml) for indicated time points. Extent of phosphorylation of β-catenin were determined in control and ETOH treated cells by Western blot analysis (top) using phospho-specific antibody against β-catenin (Ser33/Ser37/Thr41). Phosphorylation of β-catenin was evaluated by normalizing with β-catenin and β-catenin expression levels were normalized using tubulin (bottom). Statistical analysis was performed using one-way ANOVA followed by Newman-Keul’s posthoc test. *denotes p<0.05 when compared with untreated control, n = 3.
(TIF)

Figure S4 **Wnt-3a inhibits basal and ETOH-induced PDCD4 protein expression.** Neuroblasts were pre-treated with recombinant Wnt-3a (25 ng/ml) for 1 h followed by treatment of ETOH (4 mg/ml) for 12 h. At the end of the experiment, lysates were immunoblotted for PDCD4 and tubulin expression. PDCD4 expression was evaluated by normalizing with tubulin. Statistical analysis was performed using one-way ANOVA followed by Newman-Keul’s posthoc test. *denotes p<0.05, n = 3.
(TIF)

Figure S5 **Effect of LiCl on GSK-3β Ser 9 phosphorylation.** SH-SY5Y cells were pretreated with or without 10 mM LiCl for 1 h followed by ETOH treatment for 12 h and were probed with anti-phospho-GSK-3β (Ser 9) and GAPDH.
(TIF)

Figure S6 **Chemical inhibition of GSK-3β with SB-216763 decreases PDCD4 protein expression in SH-SY5Y.** Cells were treated with or without SB-216763 (20 µM) for 12 h and were immunoblotted for PDCD4 and GAPDH. Statistical significance was evaluated by Student’s t test. *denotes p<0.05 compared with control. n = 3.
(TIF)

Figure S7 **Chemical inhibition of GSK-3β with SB-216763 decreases PDCD4 protein expression in differentiated SH-SY5Y.** **(A)** Depicts retinoic acid (RA)-induced differentiation of SH-SY5Y cells. SH-SY5Y cells were treated with RA (1 µM) for 48 h and images were taken at 20X objective using transmitted light inverted imaging system (Advanced Microscopy Group, Evos XL Cell Imaging System). Prominent neurites are evident suggesting differentiation. **(B)** Representative Western image of neuronal marker neurofilament-200 (NF-200) expression confirming successful differentiation. **(C)** RA-differentiated SH-SY5Y cells were treated with or without SB-216763 (20 µM) for 6 h and were immunoblotted for PDCD4 and GAPDH. Inhibition of GSK-3β blocked basal PDCD4 expression in differentiated neurons. Statistical significance was evaluated by Student’s t test. *denotes p<0.05 compared with control. n = 3.
(TIF)
