# Supplementary figures and images for: Ethanol-Induced Transcriptional Activation of Programmed Cell Death 4 (Pdcd4) Is Mediated by GSK-3β Signaling in Rat Cortical Neuroblasts
Source: PLoS One. 2014 May 16;9(5):e98080. doi: 10.1371/journal.pone.0098080 (PMC4024002; doi:10.1371/journal.pone.0098080)

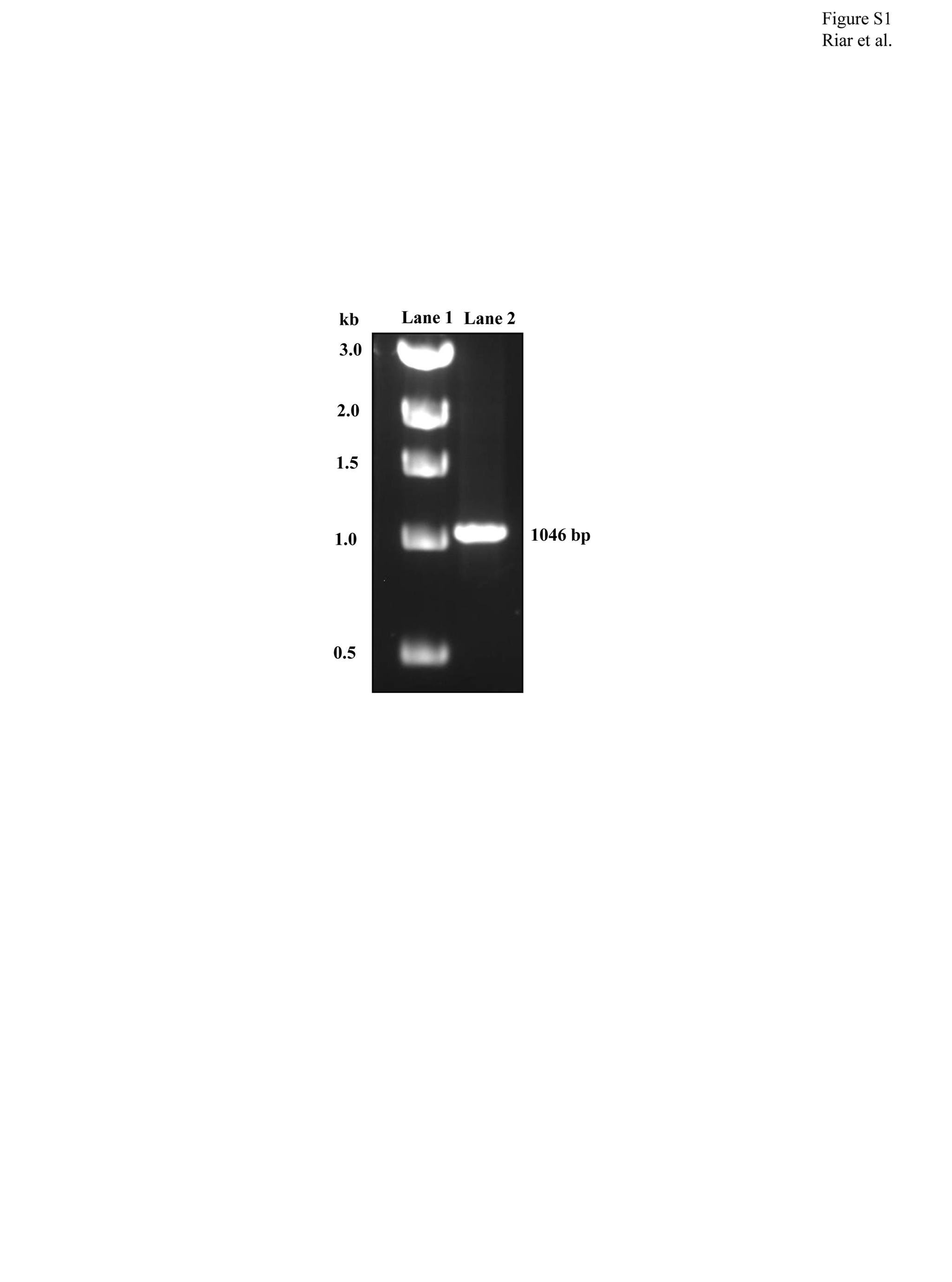

Supplement: Figure S1 — Amplification of −1046 bp rat Pdcd4 promoter fragment. Agarose gel electrophoresis showing the amplification of putative Pdcd4 promoter fragment of 1046 (PD PROM) using the primers described in methods section. PCR product was resolved in 1% agarose gel and visualized by staining with ethidium bromide. Lane 1 and lane 2 depicts 1 kb ladder and PCR amplification product respectively. (TIF) [file pone.0098080.s001.tif]

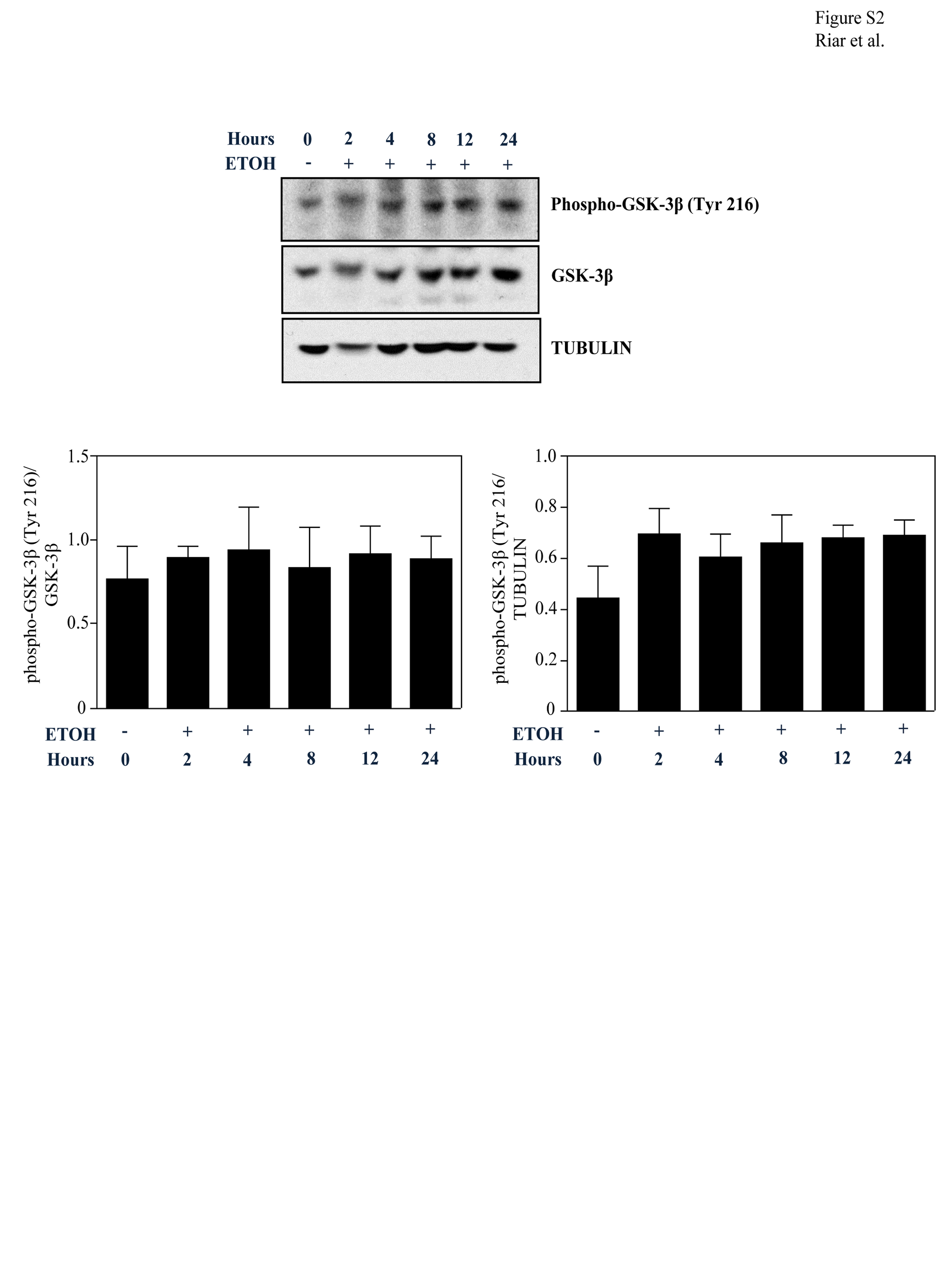

Supplement: Figure S2 — Effect of ethanol on GSK-3β Tyr 216 phosphorylation. Neuroblasts were treated with ETOH (4 mg/ml) for 2, 4, 8, 12 and 24 h. Tyrosine phosphorylation of GSK-3β was determined in control and ETOH treated cells by Western blot analysis using p-GSK3Tyr 216 specific antibody (top). Statistical significance was evaluated by normalizing with GSK-3β and tubulin (bottom). Statistical analysis was performed using one-way ANOVA followed by Newman-Keul’s posthoc test. Datapoints were not significant when compared to untreated control, n = 3. (TIF) [file pone.0098080.s002.tif]

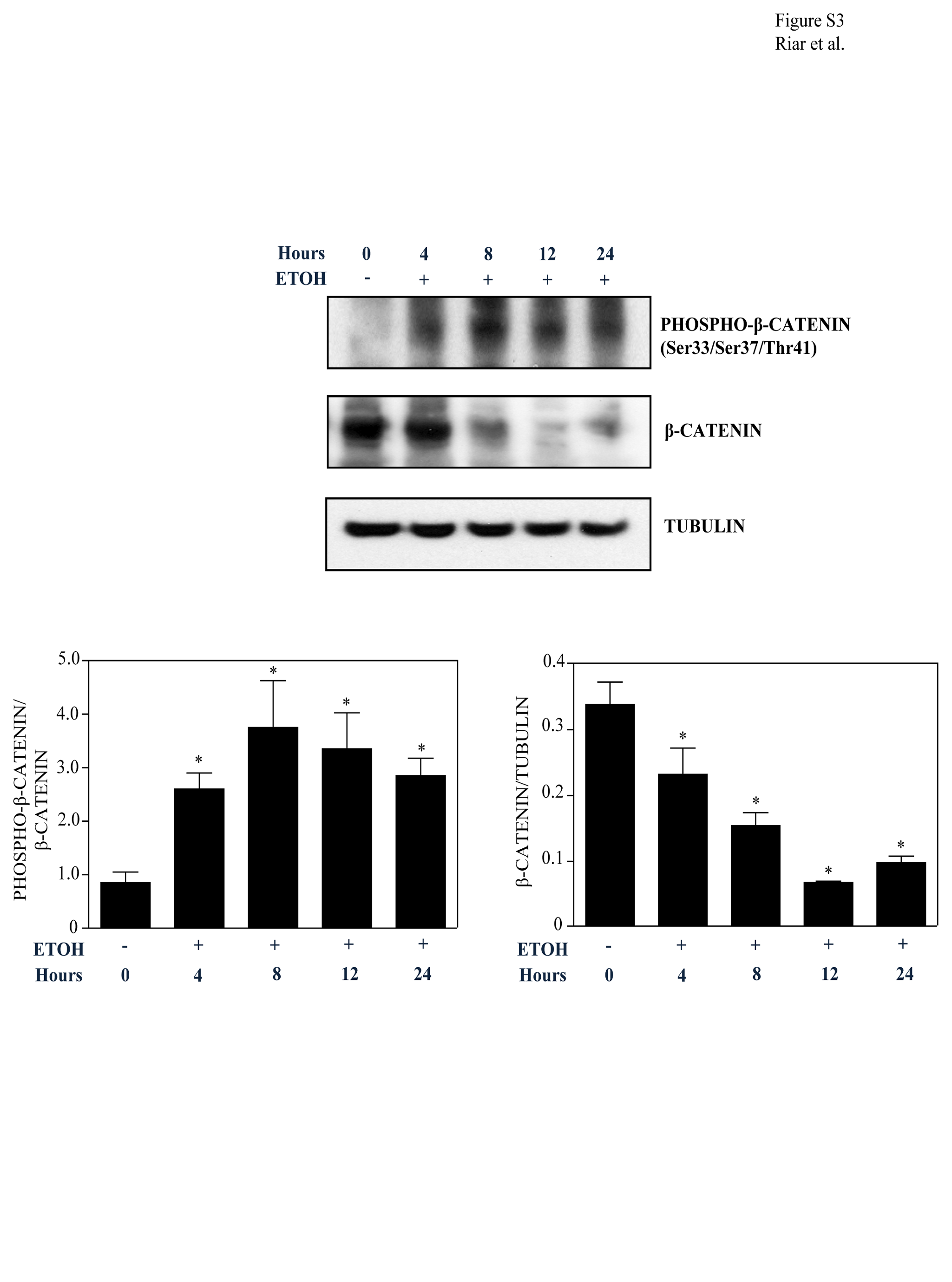

Supplement: Figure S3 — Ethanol enhances phosphorylation and degradation of β-catenin. Neuroblasts were treated with ETOH (4 mg/ml) for indicated time points. Extent of phosphorylation of β-catenin were determined in control and ETOH treated cells by Western blot analysis (top) using phospho-specific antibody against β-catenin (Ser33/Ser37/Thr41). Phosphorylation of β-catenin was evaluated by normalizing with β-catenin and β-catenin expression levels were normalized using tubulin (bottom). Statistical analysis was performed using one-way ANOVA followed by Newman-Keul’s posthoc test. *denotes p<0.05 when compared with untreated control, n = 3. (TIF) [file pone.0098080.s003.tif]

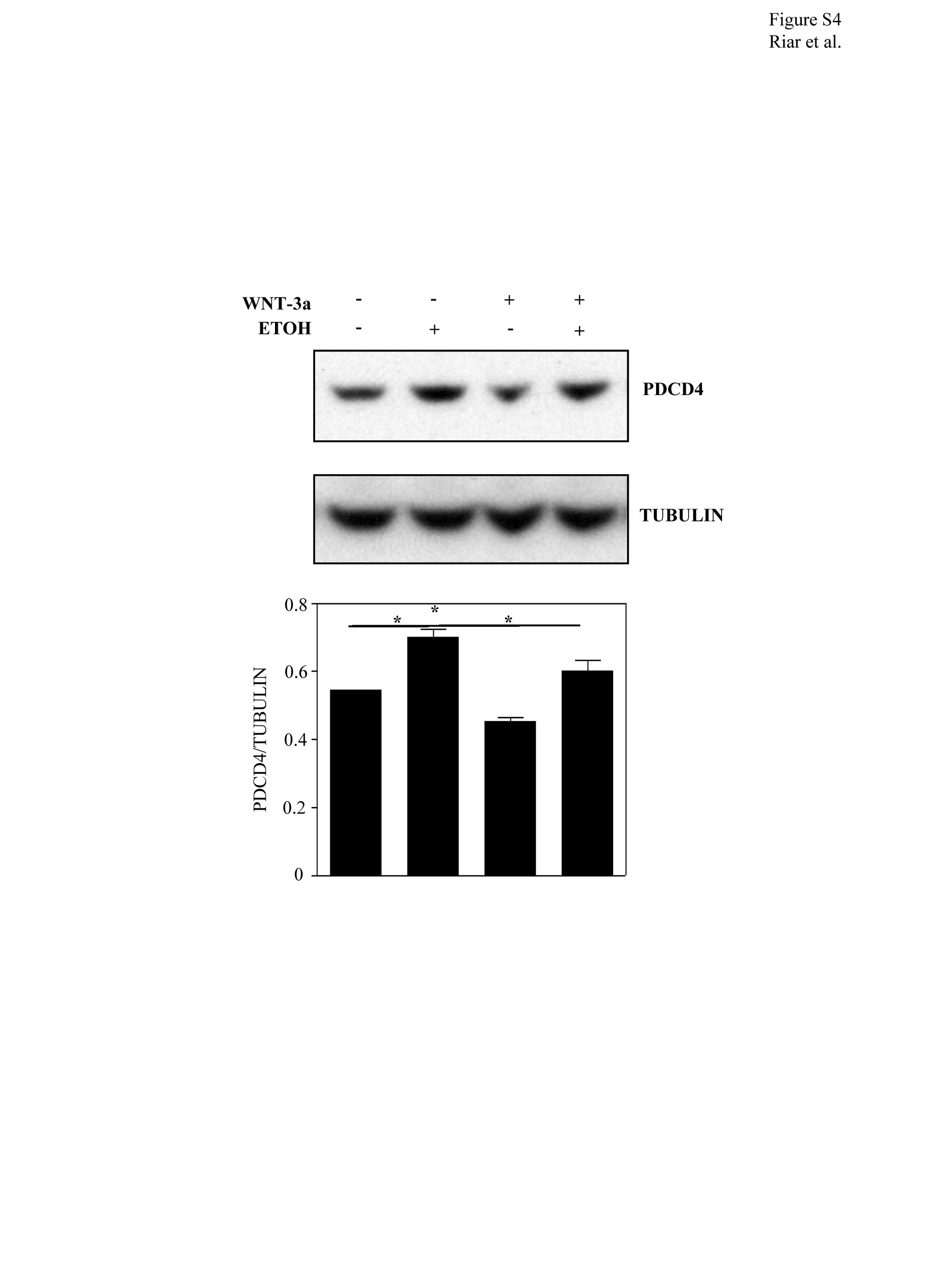

Supplement: Figure S4 — Wnt-3a inhibits basal and ETOH-induced PDCD4 protein expression. Neuroblasts were pre-treated with recombinant Wnt-3a (25 ng/ml) for 1 h followed by treatment of ETOH (4 mg/ml) for 12 h. At the end of the experiment, lysates were immunoblotted for PDCD4 and tubulin expression. PDCD4 expression was evaluated by normalizing with tubulin. Statistical analysis was performed using one-way ANOVA followed by Newman-Keul’s posthoc test. *denotes p<0.05, n = 3. (TIF) [file pone.0098080.s004.tif]

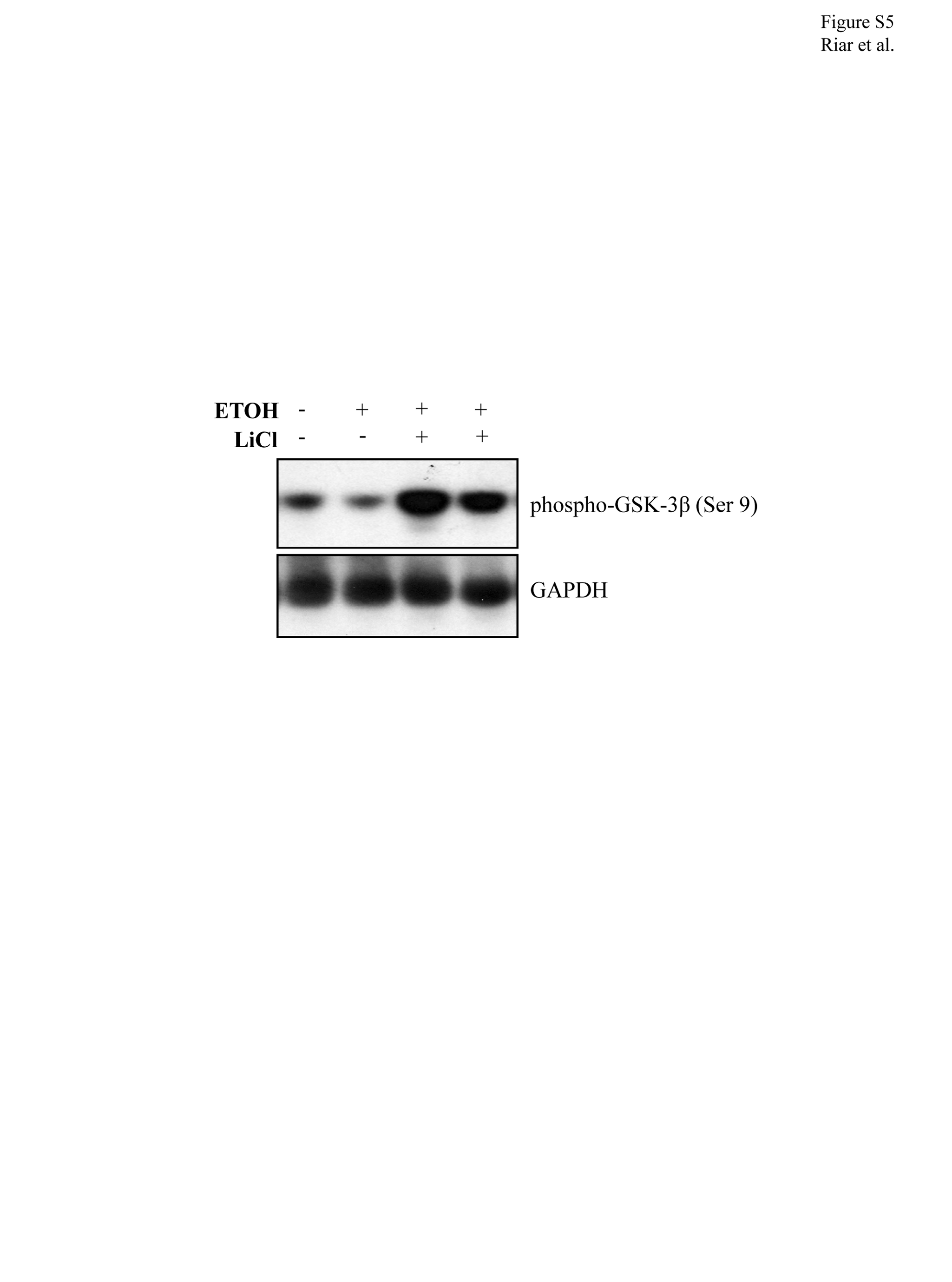

Supplement: Figure S5 — Effect of LiCl on GSK-3β Ser 9 phosphorylation. SH-SY5Y cells were pretreated with or without 10 mM LiCl for 1 h followed by ETOH treatment for 12 h and were probed with anti-phospho-GSK-3β (Ser 9) and GAPDH. (TIF) [file pone.0098080.s005.tif]

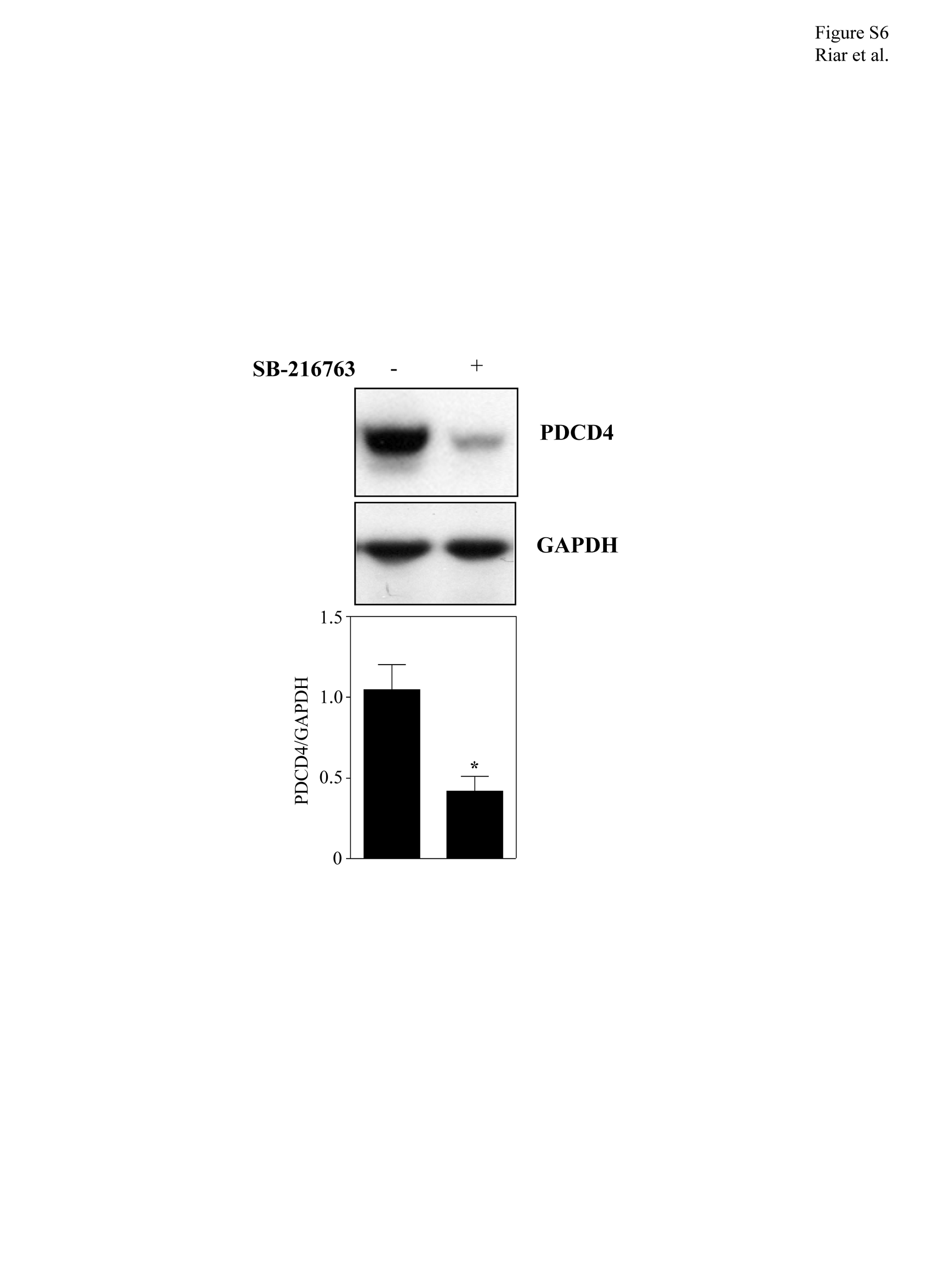

Supplement: Figure S6 — Chemical inhibition of GSK-3β with SB-216763 decreases PDCD4 protein expression in SH-SY5Y. Cells were treated with or without SB-216763 (20 µM) for 12 h and were immunoblotted for PDCD4 and GAPDH. Statistical significance was evaluated by Student’s t test. *denotes p<0.05 compared with control. n = 3. (TIF) [file pone.0098080.s006.tif]

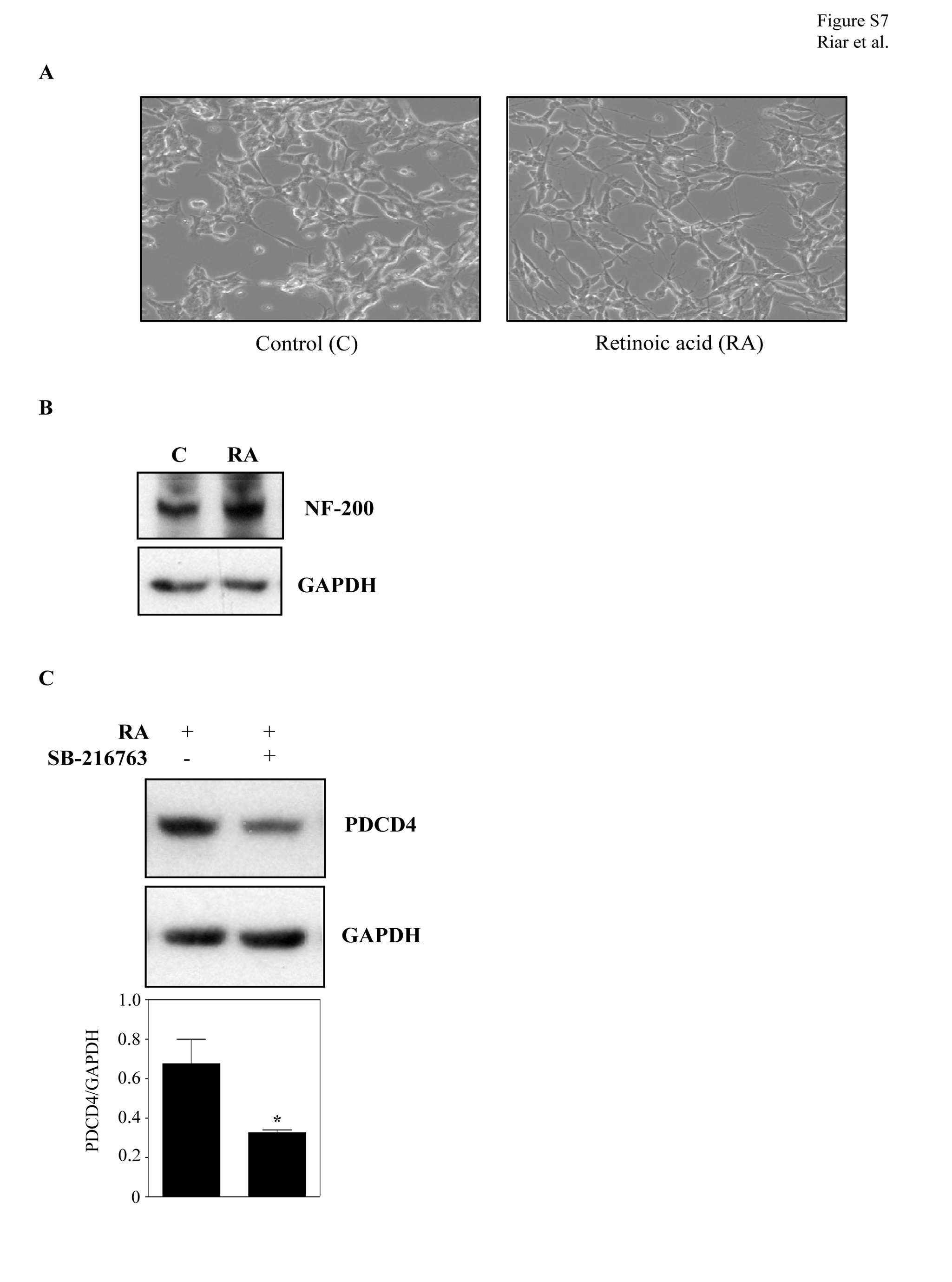

Supplement: Figure S7 — Chemical inhibition of GSK-3β with SB-216763 decreases PDCD4 protein expression in differentiated SH-SY5Y. (A) Depicts retinoic acid (RA)-induced differentiation of SH-SY5Y cells. SH-SY5Y cells were treated with RA (1 µM) for 48 h and images were taken at 20X objective using transmitted light inverted imaging system (Advanced Microscopy Group, Evos XL Cell Imaging System). Prominent neurites are evident suggesting differentiation. (B) Representative Western image of neuronal marker neurofilament-200 (NF-200) expression confirming successful differentiation. (C) RA-differentiated SH-SY5Y cells were treated with or without SB-216763 (20 µM) for 6 h and were immunoblotted for PDCD4 and GAPDH. Inhibition of GSK-3β blocked basal PDCD4 expression in differentiated neurons. Statistical significance was evaluated by Student’s t test. *denotes p<0.05 compared with control. n = 3. (TIF) [file pone.0098080.s007.tif]
